# The thrombopoietin receptor, c-Mpl, is a selective surface marker for human hematopoietic stem cells

**DOI:** 10.1186/1479-5876-4-9

**Published:** 2006-02-16

**Authors:** John M Ninos, Leigh C Jefferies, Christopher R Cogle, William G Kerr

**Affiliations:** 1 H. Lee Moffitt Cancer Center and Research Institute, Immunology Program, Department of Interdisciplinary Oncology, University of South Florida, SRB-2, 12902 Magnolia Drive, Tampa, FL 33612-9416, USA; 2 AstraZeneca LP, Drug Safety US, FOC NW2-263, Wilmington, Delaware 19850-5437, USA; 3 University of Florida, Division of Hematology/Oncology, 1600 SW Archer Road, ARB R4-252, P.O. Box 100277, Gainesville, FL 32610-0277, USA; 4 H. Lee Moffitt Cancer Center and Research Institute, Immunology Program, Departments of Interdisciplinary Oncology and Biochemistry, University of South Florida, SRB-2, 12902 Magnolia Drive, Tampa, FL 33612-9416, USA

## Abstract

**Background:**

Thrombopoietin (TPO), the primary cytokine regulating megakaryocyte proliferation and differentiation, exerts significant influence on other hematopoietic lineages as well, including erythroid, granulocytic and lymphoid lineages. We previously demonstrated that the receptor for TPO, c-mpl, is expressed by a subset of human adult bone marrow hematopoietic stem/progenitor cells (HSC/PC) that are enriched for long-term multilineage repopulating ability in the SCID-hu Bone *in vivo *model of human hematopoiesis.

**Methods:**

Here, we employ flow cytometry and an anti-c-mpl monoclonal antibody to comprehensively define the surface expression pattern of c-mpl in four differentiation stages of human CD34^+ ^HSC/PC (*I*: *CD34*^+^*38*^--^, *II*: *CD34*^+^*38*^*dim*^, *III*: *CD34*^+^*38*^+^, *IV*: *CD34*^*dim*^*38*^+^) for the major sources of human HSC: fetal liver (FL), umbilical cord blood (UCB), adult bone marrow (ABM), and cytokine-mobilized peripheral blood stem cells (mPBSC). We use a surrogate *in vivo *model of human thymopoiesis, SCID-hu Thy/Liv, to compare the capacity of c-mpl^+ ^vs. c-mpl^-- ^CD34^+^38^--/dim ^HSC/PC for thymocyte reconstitution.

**Results:**

For all tissue sources, the percentage of c-mpl^+ ^cells was significantly highest in stage *I *HSC/PC (FL 72 ± 10%, UCB 67 ± 19%, ABM 82 ± 16%, mPBSC 71 ± 15%), and decreased significantly through stages *II*, *III*, and *IV *((FL 3 ± 3%, UCB 8 ± 13%, ABM 0.6 ± 0.6%, mPBSC 0.2 ± 0.1%) [ANOVA: P < 0.0001]. The relative median fluorescence intensity of c-mpl expression was similarly highest in stage *I*, decreasing through stage *IV *[ANOVA: P < 0.0001]. No significant differences between tissue sources were observed for either % c-mpl^+ ^cells [P = 0.89] or intensity of c-mpl expression [P = 0.21]. Primary Thy/Liv grafts injected with CD34^+^38^--/dim^c-mpl^+ ^cells showed slightly higher levels of donor HLA^+ ^thymocyte reconstitution vs. CD34^+^38^--/dim^c-mpl^--^-injected grafts and non-injected controls (c-mpl^+ ^vs. c-mpl^--^: CD2^+ ^6.8 ± 4.5% vs. 2.8 ± 3.3%, CD4^+^8^-- ^54 ± 35% vs. 31 ± 29%, CD4^--^8^+ ^29 ± 19% vs. 18 ± 14%).

**Conclusion:**

These findings support the hypothesis that the TPO receptor, c-mpl, participates in the regulation of primitive human HSC from mid-fetal through adult life. This study extends our previous work documenting human B-lineage, myeloid and CD34^+ ^cell repopulation by c-mpl^+ ^progenitors to show that c-mpl^+ ^HSC/PC are also capable of significant T-lineage reconstitution *in vivo*. These results suggest that c-mpl merits consideration as a selective surface marker for the identification and isolation of human HSC in both basic research and clinical settings.

## Background

Elucidating the nature and biology of the primitive pluripotent hematopoietic stem cell (HSC) has been a major goal of research efforts in hematopoiesis. Practical and accurate identification of primitive human HSC is useful for both research investigation of stem cell physiology, as well as for clinical applications, such as stem cell transplantation, stem cell expansion and gene therapy. Isolation of candidate human HSC allow investigations into stem cell renewal, expansion, and key events in lineage commitment during maturation and development. Positive selection strategies allow transplantation of HSC capable of long-term multilineage hematopoietic reconstitution, while avoiding transfer of cells responsible for graft versus host disease in the allogeneic setting, or contaminating tumor cells in the autologous setting.

It is now common practice to utilize monoclonal antibodies raised against an array of relevant cell surface antigens for the identification and purification of subpopulations of human hematopoietic stem/progenitor cells (HSC/PC) [1]. Historically, the surface marker most commonly used for human HSC/PC isolation has been the CD34 antigen [2,3]. Human hematopoietic cells expressing the CD34 antigen are enriched for HSC/PC, but represent a heterogeneous population, including early progenitors with high levels of CD34 (CD34^Bright^) [4], and lineage-committed progenitors, with decreasing levels of CD34 as they differentiate (CD34^dim^) [1,5,6]. The long-term multipotent repopulating capability of CD34^+ ^HSC/PC, which encompasses B- and T-lymphoid, myelomonocytic, erythroid, and megakaryocytic reconstitution *in vivo*, resides within the CD34^Bright ^fraction [4]. In order to more finely delineate those CD34^Bright ^cells which possess the reconstitutive and self-renewal properties of primitive HSC, other cell surface antigens have been investigated, such as CD38, CD50, HLA-DR, CD71, CD90, CD117, and more recently CD133 [1] and CDCP1 [7,8].

The CD38 antigen has been useful to further identify CD34^+ ^HSC/PC that have begun the initial stages of lineage commitment. CD38 is a 45 kD glycoprotein whose function is unknown. Terstappen et al. [6] found that expression of CD38 is an early event in the differentiation of human CD34^+ ^adult bone marrow (ABM) cells into the erythroid, myeloid and lymphoid lineages, and therefore, a useful marker for indicating early lineage commitment. Based on the differential expression of the CD34 and CD38 differentiation antigens, the lineage-associated antigens CD71, CD33, CD10, CD5 and CD7, together with forward and orthogonal light-scattering properties, the authors developed a model for human ABM HSC/PC differentiation to include four progressive developmental stages I through IV. Stages I (CD34^++^CD38^--^) and II (CD34^++/+^CD38^+/++^) included the uncommitted, multipotent progenitor cells; stages III (CD34^+^CD38^+++^) and IV (CD34^dim^CD38^+++^) included the lineage-committed progenitor cells. Subsequent studies have confirmed the basic tenets of this model, and shown that the CD34^+^CD38^-- ^stage I HSC/PC are highly enriched for pluripotent stem cell activity [9], including long-term culture-initiating cells (LTC-IC) and long-term repopulating stem cells [5,6,10-12].

Many studies of HSC have identified factors that influence maintenance or self-renewal of the stem cell and factors that lead to differentiation and lineage commitment. Thrombopoietin (TPO) is a cytokine isolated by several groups and determined to be the primary regulator of lineage-committed megakaryocyte and platelet development [13]. However, evidence has accumulated to indicate that TPO also exerts significant influence on other hematopoietic lineages as well [14-16]. The question remained whether TPO was affecting a broad array of lineage-committed progenitor cells, or alternatively, acting on earlier multipotent progenitors and primitive pluripotent stem cells.

TPO interacts with the surface receptor, c-mpl, a member of the hematopoietic growth factor receptor superfamily [13]. Vigon et al. [17] originally cloned and characterized c-*mpl *as the human homolog of the v-*mpl *oncogene from the cDNA library of the human erythroid leukemia cell line. They noted its expression by reverse transcriptase-polymerase chain reaction (RT-PCR) in human placenta, bone marrow (BM), fetal liver (FL), fetal peripheral blood, umbilical cord blood (UCB) and adult peripheral blood (PB), and by RNA blot in murine immature hematopoietic precursor cells [18]. Methia et al. [19] found that c-*mpl *mRNA transcripts were expressed in purified CD34^+ ^hematopoietic cells by RT-PCR. We subsequently found that c-mpl is expressed at the cell surface by a subset of murine and human HSC/PC that are enriched for cells capable of long-term multilineage hematopoietic repopulation in primary recipients [20].

In the current study, we further define the expression of the c-mpl surface receptor in human CD34^+ ^HSC/PC isolated from four major sources of HSC from midfetal through adult life: FL, UCB, ABM, and cytokine-mobilized peripheral blood stem cells (mPBSC). This analysis reveals that c-mpl receptor expression is highest on the most primitive Stage I subset of CD34^+ ^HSC/PC and progressively declines through Stages II, III and IV, regardless whether the source is fetal, neonatal or adult. Moreover, the intensity of c-mpl expression can serve to define a primitive stage of human CD34^+ ^HSC/PC from these four tissue sources. In addition, we show that the c-mpl^+ ^fraction, in contrast to the c-mpl^-- ^fraction, shows a significant increase in T-lineage thymocyte repopulation versus control grafts in the SCID-hu Thy/Liv *in vivo *model of human thymic development. These studies further indicate that c-mpl is a selective human HSC surface receptor with a relevant role in the regulation of the stem cell compartment, and have practical implications for HSC positive selection, expansion, and transplantation protocols for both basic research and clinical applications.

## Methods

### Human tissues

Anti-coagulated UCB samples were obtained from two sources. UCB specimens were collected at the University of Pennsylvania Health System Cord Blood Program (Philadelphia, PA) and the Life South Cord Blood Bank of the University of Florida (Gainesville, FL) with appropriate informed consents granted by the patients. Fresh ABM mononuclear cell specimens were obtained from Poietic Technologies (Gaithersburg, MD) from normal healthy volunteers with full informed consent. mPBSC were obtained from four cancer patients who were mobilized with a standard regimen of G-CSF (Neupogen) beginning 5 days prior to collection and twice daily during collection. mPBSC collections were performed by the University of Pennsylvania Transfusion Medicine Section using either COBE Spectra (Gambro BCT, Inc., Lakewood, CO) or Fenwal CS-3000 Plus (Baxter Healthcare Co., Deerfield, IL) blood cell separators. The four patients were being treated with high dose chemotherapy followed by mPBSC transplant for the following diseases: Hodgkin's Disease, Neuroblastoma, and Non-Hodgkin's Lymphoma (2 patients). All patients consented to the use of small aliquots of these cells for research purposes according to the University of Pennsylvania Hospital and Pathology Department guidelines effective at the time of collection. Human fetal tissues (liver, thymus and femurs) were obtained from Advanced Bioscience Resources (Alameda, CA) with all appropriate and necessary informed consent measures fulfilled.

### Flow cytometric analysis of human HSC

Mononuclear cells (MNC) from FL, UCB, ABM and mPBSC were isolated, counted on a hemacytometer, pelleted at 200 g for 10 minutes at 4°C and resuspended in 300 ul of chilled, degassed MACS^® ^Buffer (MB) [phosphate buffered saline pH 7.2, 0.5% bovine serum albumin, 2 mM EDTA] per 10^8 ^cells. Prior to antibody staining, the MNC were FcR-blocked with human IgG, then magnetically labeled with anti-CD34 coupled indirectly to MACS^® ^magnetic microbeads using the Miltenyi CD34 Progenitor Cell isolation kit (Miltenyi Biotech, Auburn, CA) according to the manufacturer's instructions. Following magnetic labeling, the labeled MNC cells were washed, resuspended in 500 uL chilled MB per 10^8 ^cells and loaded onto a Miltenyi MACS^® ^VS+ positive selection column and placed in the magnetic field of a Vario MACS^® ^separator. The column was washed 3× with chilled MB. The CD34^+ ^cells remaining in the column were eluted with 5 ml of chilled MB. The CD34^+ ^selected cells were counted on a hemacytometer and stained with the following two antibody panels: 1) isotype control panel: CD34-FITC (clone 581; 10 uL; BD Biosciences, San Diego, CA), CD38-PE (clone HIT2; 3 uL; Caltag Laboratories, Burlingame, CA) and mouse IgG1-biotin (2.4 ug/24 uL; Caltag) and 2) c-mpl panel: CD34-FITC, CD38-PE, c-mpl-biotin (clone 3G4/CD110; 2.4 ug/5 uL; Genentech, Inc., San Franciso, CA) [21,22]. Biotinylated antibodies were revealed with Streptavidin-APC (3 uL/10^6 ^cells; BD Biosciences). The final concentration of IgG1-biotin and c-mpl-biotin for each experiment were equivalent. The cells were stained at a concentration of 10^6 ^cells/50 uL staining medium (SM) [PBS, 1% fetal bovine serum] for 30 minutes at 4°C. The cells were washed in SM, pelleted at 300 g and resuspended in SM with either propidium iodide (PI) [1 ug/mL; Sigma, St. Louis, MO] or 7-AAD (10 uL/mL; BD Biosciences) for dead cell exclusion. The stained, CD34-selected cells were then acquired on either a FACSVantage™ or a FACSCalibur™ flow cytometer using CellQuest™ software (BD Biosciences). Data was analyzed using FlowJo^® ^version 4 software (Treestar Inc., Ashland, Oregon). From both staining panels, 100,000 events were acquired, unless sample size was limiting (UCB: 7,000–38,000 events; FL 40,000–100,000 events). The analysis protocol was consistent and uniform across all samples from each HSC source. Minor adjustments to the gates were made between individual specimens to account for experimental variability. Beginning with the cells stained with the isotype control panel, a "*cell*" gate was created on a forward scatter (FSC) vs. side scatter (SSC) plot to eliminate debris, platelets, red cells, cell doublets, and MNC with high SSC. From within the "*cell*" gate, a "*viability*" gate was applied on a log histogram plot of PI or 7-AAD to exclude nonviable events. A CD34-FITC vs. CD38-PE contour plot was then selected on viable cells. From this plot, four HSC/PC differentiation stage subsets based on CD34 vs. CD38 expression were delineated as per Terstappen *et al*. [6]: (*I*): *CD34*^+^*CD38*^-- ^(*II*): *CD34*^+^*CD38*^dim ^(*III*): *CD34*^+^*CD38*^+ ^(*IV*): *CD34*^dim^*CD38*^+^. From within each "*CD34/CD38 stage*" of the isotype control-stained cells, an IgG1-APC histogram plot was created. The "*c-mpl*^--^" gate was then defined by including 99.5% of the IgG1-APC negative events. This gate was adjusted by ± 0.1–0.2% when the FlowJo software could not select exactly 99.5% of the events. Conversely, a "*c-mpl*^+^" gate was defined to include 0.5% (± 0.1–0.2%) of the IgG1-APC positive events and extended to the far right of the APC histogram. The "*cell*", "*viability*", "*CD34/CD38 stage*" and "*c-mpl*^+^" gates were then applied exactly, without alteration, to the paired cell sample stained with the c-mpl antibody panel to determine the percentage of c-mpl^+ ^cells in each *CD34/CD38 stage*. Two statistical methods in the FlowJo software – super-enhanced Dmax subtraction (SED) [23] and Overton subtraction [24] – were also used to determine the percentage of c-mpl^+ ^events relative to the isotype control in a univariate population comparison. Furthermore, the median fluorescence intensity (MFI) of the log APC parameter for each *CD34/CD38 stage *from each specimen was determined for both the IgG1 isotype control and c-mpl panel-stained sample pairs. The MFI of the c-mpl panel-stained cells was then normalized against the MFI of the IgG1 isotype control panel-stained cells for each *CD34/CD38 *subset to allow comparison of the Relative MFI (RMFI) across each specimen and each stem cell tissue source. Next, for each sample from every tissue source, we again selected those events falling within both the "*cell*" gate and the "*viable*" gate to include only viable MNC. We analyzed these viable MNC for c-mpl-APC fluorescence intensity on a histogram plot of the log APC parameter. We then selected three discrete subsets of cells based solely on their percentile level of c-mpl-APC fluorescence intensity for further analysis: (1) High [98–100%^tile^] (2) Intermediate [48–50%^tile^] and (3) Low [18–20%^tile^]. These three percentile groups were then analyzed on CD34-FITC vs. CD38-PE contour and dot plots to discern qualitative differences in CD34 and CD38 expression. A similar anaysis was then performed on a select number of specimens for a more complete range of High percentile groupings by drawing five gates on the histogram plot to define a progression of five discrete levels of High c-mpl expression: 79–80%^tile^, 84–85%^tile^, 89–90%^tile^, 94–95%^tile^, and 99–100%^tile^.

Statistical comparisons were performed using Prism 4.0 software (GraphPad Software, San Diego, CA). By convention, the threshold value, α, was set to 0.05. The percentage of viable c-mpl^+ ^cells was determined by: 1) manual gating, 2) the SED algorithm, and 3) the Overton subtraction algorithm. For each human tissue studied (FL, UCB, ABM, mPBSC), the following human *HSC/PC *subset groups were compared in a repeated measures two-way analysis of variance: (*I*) *CD34*^+^*CD38*^--^, (*II*) *CD34*^+^*CD38*^*dim*^, (*III*) *CD34*^+^*CD38*^+^, and (*IV*) *CD34*^*dim*^*CD38*^+^. Tukey's and Bonferroni's Multiple Comparison post-tests were then performed to compare pairs of group means. In addition, a post-test for a linear trend was performed with the subset groups arranged in the following order: *I*, *II*, *III*, *IV*. Next, the RMFI values (after normalizing to the isotype control MFI) were analyzed in the same manner and using the same groups for comparison as for the c-mpl^+ ^analyses described above.

### Generation of SCID-*hu *Thy/Liv mice

Eight week old C.B-17 *scid/scid *severe combined immune deficiency (SCID) mice were implanted with human fetal thymus and liver fragments as previously described [25-28]. Briefly, human fetal thymus and liver tissue were obtained from 18–22 week gestational fetuses. During the preparation of the tissue grafts, MNC were collected and analyzed by flow cytometry using a panel of human leukocyte antigen (HLA) antibodies (One Lambda, Inc., Canoga Park, CA) and found to be negative for HLA-B8. The 8 week old SCID mice were anesthetized with Ketamine. The fur over the mouse kidney was shaved and the skin prepped with alcohol and betadine. The skin and peritoneum were opened over the right kidney. The kidney was exposed with a hemostat. Two ~1 mm^3 ^cubes of fetal liver were placed adjacent to one ~1 mm^3 ^cube of fetal thymus in a 16 gauge trocar and co-implanted as a unit under the renal capsule. The peritoneal and fascial layers were restored with sutures (Ethicon, Somerville, NJ), and the skin incision was secured with MikRon 9 mm Autoclips (MikRon Precision, Gardena, CA). The human Thy/Liv transplants were allowed to engraft for 8 weeks.

### Analysis of engraftment in SCID-*hu *Thy/Liv grafts

Bone marrow MNC from an HLA-B8^+ ^normal volunteer donor were positively selected for the CD34 antigen with CD34 immunolabeled magnetic beads using the CD34 Progenitor Cell kit (Miltenyi Biotech). The CD34^+ ^cells were stained with CD34-FITC, CD38-PE, and biotinylated anti-c-mpl (clone 3G4/CD110) [21,22]. A small aliquot of the sample was stained with CD34-FITC, CD38-PE and biotinylated IgG1-isotype control. The biotinylated antibodies were revealed with Streptavidin-APC. The cells were washed and resuspended in SM containing PI (1 ug/mL). Samples were acquired and sorted on a FACSVantage™ flow cytometer using CellQuest™ software. After gating for viable cells based on PI exclusion and light side scatter, an IgG1-APC histogram for CD34^+^CD38^--/dim ^cells was plotted. A c-mpl^-- ^gate was drawn to include 99% of the cells in the IgG1 isotype control stain. A c-mpl^+ ^gate was drawn from the end of the c-mpl^-- ^gate to the far right of the histogram. These two gates were then applied to the c-mpl-panel stained cells, and CD34^+^CD38^--/dim^c-mpl^+ ^and CD34^+^CD38^--/dim^c-mpl^-- ^cells were sorted into tubes containing RPMI1640 (Mediatech, Inc., Herndon, VA) with 3% fetal bovine serum and 10 mM HEPES. The sorted cells were counted on a hemacytometer, pelleted at 200 g for 10 minutes at 4°C and resuspended in sterile PBS. The diluted CD34^+^CD38^--/dim^c-mpl^+ ^and CD34^+^CD38^--/dim^c-mpl^-- ^cells were loaded separately into a Hamilton syringe. SCID-*hu *Thy/Liv mice were irradiated with 400 rads from a Cs^137 ^source prior to injection. The mice were anesthetized with ketamine, and the Thy/Liv grafts were gently exteriorized and inspected. Only mice with healthy, robust grafts (numbering 22) were chosen for HSC subset injections. Grafts were injected with the viable sorted HSC subsets at 30,000 or 60,000 cells per graft. Ten grafts were injected with 60 k (3 grafts) or 30 k (7 grafts) of viable CD34^+^CD38^--^c-mpl^-- ^cells. Nine grafts were injected with 60 k (2 grafts) or 30 k (7 grafts) of viable CD34^+^CD38^--^c-mpl^+ ^cells. Three grafts were reserved as non-injected controls. The c-mpl^+ ^and c-mpl^--^-injected ABM stem cell subsets were then allowed to repopulate the Thy/Liv grafts for 8 weeks. After 8 weeks, surviving mice were: c-mpl^+^: 60 k (1), 30 k (3); c-mpl^--^: 60 k (2), 30 k (5); non-injected: (3). The Thy/Liv grafts from the surviving mice were carefully exteriorized and removed. Single cell suspensions from each graft were prepared and stained separately with: (1) IgG1-FITC (5 uL/10^6 ^cells; BD Pharmingen), IgG1-PE (5 uL/10^6 ^cells; BD Pharmingen), IgG2b-biotin (10 uL/10^6 ^cells; Caltag), (2) CD34-FITC (10 uL/10^6 ^cells; BD Pharmingen; clone 581), CD2-PE (10 uL/10^6 ^cells; BD Pharmingen; clone RPA-2.10) and HLA-B8-biotin (5 uL/10^6 ^cells; One Lambda), and (3) CD4-FITC (10 uL/10^6 ^cells; BD Pharmingen; clone RPA-T4), CD8-PE (10 uL/10^6 ^cells; BD Pharmingen; clone RPA-T8), and HLA-B8-biotin (5 ul/10^6 ^cells). Biotin conjugates were revealed with Streptavidin-APC (3 uL/10^6 ^cells; BD Pharmingen). Cells were resuspended in SM with PI (1 ug/mL) for dead cell exclusion. For each antibody panel, 100,000 events were acquired on a FACSCalibur™ flow cytometer using CellQuest™ software. Analysis was performed using FlowJo^® ^version 4 software. A "*human cell*" gate was drawn around the human lymphoid cell population based on a FSC vs. SSC plot. A "*viable*" gate was created around viable cells on a PI vs. FSC plot. The viable human lymphoid cell population in the non-injected Thy/Liv grafts was used to define the HLA-B8 positive and negative gates for both the CD34/CD2/HLA-B8 and CD4/CD8/HLA-B8 antibody stain panels. In each case, an HLA-B8^-- ^gate was defined to include 99.0% of the HLA-B8-stained non-injected Thy/Liv grafts, and an HLA-B8^+ ^gate was drawn to extend from the HLA-B8^-- ^gate to the extreme right of the APC parameter log histogram plot. Subsequent analysis (data not shown) confirmed that this strategy for defining the HLA-B8^+ ^events was more restrictive than the strategy of defining HLA-B8^+ ^events using an IgG2b-APC isotype control.

For statistical comparisons of Thy/Liv reconstitution, the threshold value, α, was set to 0.05. The following thymocyte subsets were analyzed: total CD2^+^, CD4^+^CD8^+^, CD4^+^CD8^--^, and CD8^+^CD4^--^. For each subset, the following three SCID-*hu *Thy/Liv graft injection groups were compared for the percentage of viable HLA-B8^+ ^lymphoid cells in a one-way analysis of variance: (1) *non-injected control *Thy/Liv grafts; (2) *CD34*^+^*CD38*^--/*dim*^*c-mpl*^--^-*injected *Thy/Liv grafts and (3) *CD34*^+^*CD38*^--/*dim*^*c-mpl*^+^-*injected *Thy/Liv grafts. A Dunnet's Multiple Comparison post-test was performed to compare separately the c-mpl^-- ^and c-mpl^+ ^injections with the non-injected control Thy/Liv grafts. In addition, a post-test for a linear trend was performed with the groups arranged in the following order: *non-injected controls*, *CD34*^+^*CD38*^--/*dim*^*c-mpl*^--^-*injections*, and *CD34*^+^*CD38*^--/*dim*^*c-mpl*^+^-*injections*.

## Results

### C-mpl is expressed by primitive HSC from all major tissue sources

**Figure 1:**
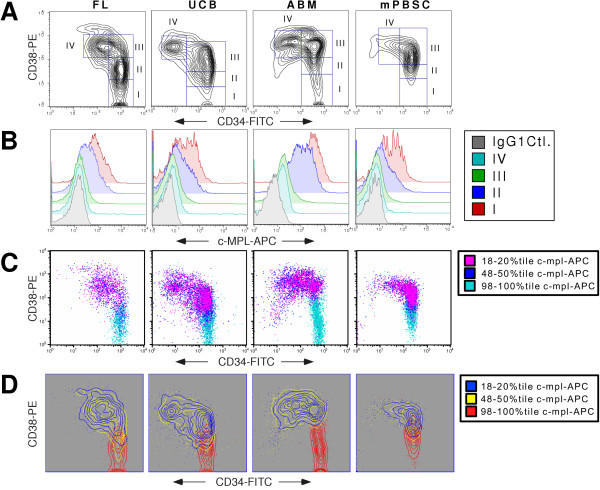
**The most primitive CD34^+ ^HSC/PC from all major tissue sources express the highest levels of c-mpl surface receptor**. **A**. Representative CD34-FITC vs. CD38-PE contour plots generated from magnetically selected viable CD34^+ ^mononuclear cells from representative specimens of the four major tissue sources of human HSC/PC: FL, UCB, ABM, and mPBSC. Gates defining the four differentiation stages of human CD34^+^HSC/PC as defined by Terstappen et al. [6] are shown: I: CD34^+^38^-^. II: CD34^+^38^dim ^III: CD34^+^38^+ ^IV: CD34^dim^38^+^. **B**. Overlay of the c-mpl-APC histogram plots of viable human CD34^+ ^mononuclear cells from the four HSC/PC differentiation stages depicted in Fig. 1A above. Stage I [red]. Stage II [blue]. Stage III [green]. Stage IV [light blue]. For comparison, a control histogram plot [gray] is shown that is generated from differentiation stage I cells stained with an IgG1 isotype control-APC antibody equivalent in concentration to the c-mpl-APC antibody stain. Isotype control histogram plots for each HSC/PC differentiation stage were used to separately set the manual gates that define the c-mpl^+ ^and c-mpl^-- ^cell populations for each stage. **C**. CD34-FITC vs. CD38-PE dot plots derived from the same representative human CD34^+ ^tissue specimens as depicted in Fig. 1A. From each specimen, all cell events were initially categorized by their percentile level of fluorescence on the c-mpl-APC parameter on a log histogram plot. Three gates were drawn on the histogram plot to define three discrete levels of c-mpl expression: High (98–100 percentile); Intermediate (48–50 percentile); Low (18–20 percentile). Viable cell events falling within one of these three percentile ranges are depicted on the dot plot with their corresponding color code: High [light blue]; Intermediate [dark blue]; Low [red]. **D**. Same data as **C**, but using contour plots to visualize the data. High [red]; Intermediate [yellow]; Low [blue].

Our previous findings demonstrated that c-mpl is expressed by the subset of CD34^+ ^cells in normal human ABM that lack or have low surface expression of CD38 [20]. The CD34^+^CD38^--/dim ^population of cells is known to be enriched for primitive HSC relative to the CD34^+^CD38^+ ^subset based on its ability to support long-term multilineage repopulation *in vivo *in a pre-immune fetal sheep model [5] or to give rise to significant numbers of LTC-IC *in vitro *[11,12]. Therefore, we conducted a more comprehensive and extensive examination of c-mpl receptor surface expression in distinct human hematopoietic CD34/CD38 HSC/PC subsets. In order to characterize more completely the expression pattern of c-mpl in human CD34^+ ^cells, we chose to investigate c-mpl expression across four different human HSC/PC differentiation stages originally characterized by Terstappen et al. [6]: (I) CD34^+^CD38^--^. (II) CD34^+^CD38^dim ^(III) CD34^+^CD38^+ ^(IV) CD34^dim^CD38^+^. We analyzed the expression of the c-mpl receptor in these four stages of HSC/PC differentiation from four major sources of human HSC: FL, UCB, ABM and mPBSC. These four tissue sources represent the major locations of human hematopoiesis from midfetal life through adulthood. In each case, we isolated CD34^+ ^cells by magnetic bead positive selection, and stained equivalent numbers of these cells with two monoclonal antibody panels: (1) Isotype control panel: monoclonal antibodies to CD34-FITC, CD38-PE and IgG1-APC or (2) c-mpl panel: monoclonal antibodies to CD34-FITC, CD38-PE and c-mpl-APC. Gates were set to identify the four stages of HSC/PC differentiation as defined by Terstappen et al. [6] (Figure 1A). In each case, the analysis was first defined by the isotype control antibody panel-stained cells, and then applied without modification to the c-mpl antibody panel-stained cells. Representative c-mpl histograms for each CD34/38 population from each tissue are shown in Figure 1B.

**Figure 2:**
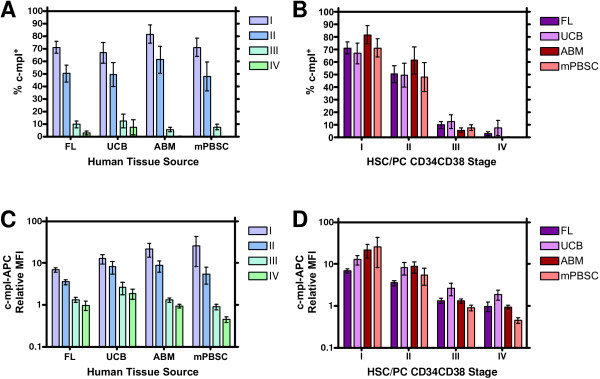
**The expression of c-mpl is significantly higher in stage I CD34^+ ^HSC/PC and decreases with advancing differentiation stage**. **A&B**. For each specimen from every tissue source, CD34^+ ^cells were plotted with FlowJo^® ^software on a CD34-FITC vs. CD38-PE contour plot, and gates demarcating HSC/PC differentiation stages I through IV [6] were applied (as depicted in Fig. 1A). Cells within each stage were stained with an IgG1 isotype control-APC antibody equivalent in concentration to the c-mpl-APC antibody used. On a log histogram plot of IgG1-APC fluorescence, a c-mpl^-- ^gate was manually drawn to include the left-most 99.5% (± 0.2%) of isotype control-stained cells. A c-mpl^+ ^gate was manually defined to extend from the end of the c-mpl^-- ^gate to the far right of the histogram plot. These gates were defined separately for each HSC/PC differentiation stage for each tissue source, and then applied without alteration to their corresponding c-mpl-APC stained cells within each stage from each tissue. The percentage of cells falling within the c-mpl^+ ^gate was then calculated by the FlowJo^® ^software. Error bars = ± standard error of mean (SEM). **C&D**. For every specimen, the median fluorescent intensity (MFI) of viable CD34^+ ^cells from each differentiation stage was calculated by FlowJo^® ^software from the log histogram plot of the c-mpl-APC fluorescence parameter. These MFI values for each stage of each tissue specimen were then normalized individually with the appropriate MFI value of the corresponding differentiation stage HSC/PCs stained with the IgG1 isotype control, yielding the Relative MFI (RMFI). Error bars = ± SEM.

Using this manual gating strategy, the percentage of c-mpl^+ ^cells in stage I was >50% for every sample tested except one (UCB#2 = 44.5%), and ranged from 44.5% to 94.5% (ABM#1) [Table 1]. For every sample tested, the percentage of c-mpl^+ ^cells decreased uniformly as the differentiation stages progressed from I through IV (Figure 2A). The mean percentage (± standard deviation [S.D.]) of c-mpl^+ ^cells in stage I ranged from 67.18% ± 18.55% (UCB) to 81.96% ± 16.29% (ABM), while the mean percentage (± S.D.) of c-mpl^+ ^cells in stage IV ranged from 0.17% ± 0.07% (PBSC) to 7.91% ± 13.43% (UCB). A repeated measures two-way analysis of variance comparing the two variables, (1) tissue source and (2) differentiation stage, indicated that the differences observed in the percentage of c-mpl^+ ^cells for the four differentiation stages were highly significant (p < 0.0001) [Table 2]. Furthermore, a post-test for a linear trend from stage I through stage IV was significant for each tissue source (P < 0.0001), indicating a significant decreasing linear trend for the percentage of c-mpl^+ ^cells successively in the order of stage I, II, III and IV. This analysis also showed that the greatest decrease in the mean percentage of c-mpl^+ ^cells between contiguous stages occurred between stages II and III for each tissue source with a mean difference ranging from 37.1% (UCB) to 55.7% (ABM). Bonferroni post-tests individually comparing pairs of contiguous stage means (I vs. II, II vs. III, III vs. IV) showed that the differences were significant for all tissue sources for stages I vs. II (p < 0.05: UCB & ABM; p < 0.01: FL & mPBSC) and stages II vs. III (p < 0.001), but not significant for stages III vs. IV. Thus, the greatest and most significant loss of c-mpl expression during this differentiation scheme is associated with the acquisition of high level CD38 expression by HSC/PC. However, there was no significant interaction between the tissue source and the differentiation stage values (P = 0.8918). Furthermore, the overall effect of the tissue source on % c-mpl^+ ^was not significant (P = 0.2949) [Figure 2B].

**Table 1:** Percentage of c-mpl^+ ^cells in human HSC/PC differentiation stages

			**PERCENTAGE c-mpl+**							
			
			**Stage I**			**Stage II**			**Stage III**	**Stage IV**
			
**Tissue Source**	**#**		**Manual Gate**	**Overton Algorithm**	**SED Algorithm**	**Manual Gate**	**Overton Algorithm**	**SED Algorithm**	**Manual Gate**	**Manual Gate**
**FL**	**5**	**Mean**	**71.54**	**77.23**	**86.43**	**50.76**	**60.19**	**72.09**	**10.15**	**3.40**
		*S.D.*	*10.48*	*8.02*	*5.94*	*14.94*	*12.04*	*11.24*	*5.99*	*3.49*
**UCB**	**5**	**Mean**	**67.18**	**76.24**	**83.82**	**49.92**	**65.83**	**75.63**	**12.85**	**7.91**
		*S.D.*	*18.55*	*11.11*	*9.55*	*21.59*	*19.74*	*18.91*	*12.51*	*13.43*
**ABM**	**5**	**Mean**	**81.96**	**85.66**	**92.25**	**61.60**	**69.87**	**80.06**	**5.93**	**0.58**
		*S.D.*	*16.29*	*13.06*	*9.69*	*23.94*	*21.76*	*19.37*	*3.93*	*0.61*
**mPBSC**	**4**	**Mean**	**71.40**	**77.58**	**85.05**	**48.25**	**57.14**	**66.85**	**7.87**	**0.17**
		*S.D.*	*14.55*	*13.25*	*9.29*	*22.86*	*19.74*	*17.39*	*4.53*	*0.07*

ABM, adult bone marrow; FL, fetal liver; mPBSC, cytokine-mobilized peripheral blood stem cells; SD, standard deviation; SED, super-enhanced Dmax subtraction; UCB, umbilical cord blood

**Table 2:** Repeated Measures Two-way ANOVA of c-mpl expression in Human HSC/PC: Stage vs. Tissue Source

	**Stage**	**Tissue**	**Interaction**	**Bonferroni's Multiple Comparison**			**Linear Trend (One-way)**
	
**% c-mpl+**	**<0.0001**	**0.8918**	**0.2949**	**Stage I vs. II**	**Stage II vs. III**	**Stage III vs. IV**	**Order: I-II-III-IV**
FL			FL	<0.01	<0.001	NS	<0.0001
UCB			UCB	<0.05	<0.001	NS	<0.0001
ABM			ABM	<0.05	<0.001	NS	<0.0001
mPBSC			mPBSC	<0.01	<0.001	NS	<0.0001

**c-mpl *RMFI***	**<0.0001**	**0.2102**	**0.0779**	**Stage I vs. II**	**Stage II vs. III**	**Stage III vs. IV**	**Order: I-II-III-IV**

FL			FL	NS	<0.05	NS	<0.0001
UCB			UCB	NS	<0.01	NS	<0.001
ABM			ABM	NS	<0.001	NS	<0.0001
mPBSC			mPBSC	<0.01	<0.001	NS	<0.0001

Values represent calculated p values for the indicated analyses. ABM, adult bone marrow; ANOVA, analysis of variance; FL, fetal liver; HSC/PC, hematopoietic stem cells/progenitor cells; mPBSC, cytokine-mobilized peripheral blood stem cells; NS, not significant (p > 0.05); RMFI, relative median fluorescence intensity; UCB, umbilical cord blood

We confirmed these findings using two mathematical comparison algorithms that provide the percentage of events that are positive compared to the control population. The Overton cumulative histogram substraction algorithm [24] was approximately 4 to 9 percentage points higher than the manual gating method for stage I, ranging from 76.24% ± 11.11% (UCB) to 85.66% ± 13.06% (ABM). The stage I mean % c-mpl^+ ^calculated by the super-enhanced Dmax subtraction (SED) algorithm [23] was approximately 10–17 percentage points higher than the manual gating method, ranging from 83.82% ± 9.55% (UCB) to 92.25% ± 9.69% (ABM). Likewise, the % c-mpl^+ ^for each sample as determined by either the Overton or SED subtraction algorithms decreased uniformly as the differentiation stages progressed from stage I through IV.

In order to quantify differences in the intensity of c-mpl receptor expression between the four differentiation stages of each sample, we calculated the MFI of c-mpl-APC staining on a histogram plot of the APC parameter using a log scale. For each differentiation stage of each sample, we similarly calculated the MFI of the isotype control-APC on the log scale histogram plot. We then divided the c-mpl MFI by the isotype control MFI to derive a Relative MFI (RMFI) normalized to the isotype control staining intensity.

In every sample except two (UCB#3 & UCB#5), the RMFI was highest in differentiation stage I and decreased uniformly through stages II, III and IV (Table 3). In UCB#3, the RMFI was slightly higher in stage II (16.69) than stage I (14.44). In UCB#5, the RMFI was slightly higher in stage IV (3.48) than stage III (2.37). The mean RMFI in stage I ranged from 7.13 ± 2.06 (FL) to 26.55 ± 36.08 (in PBSC). The mean RMFI for each tissue source decreased uniformly from differentiation stage I through stages II, III and IV (Figure 2C). Because these data were derived from a logarithmic scale, the values were transformed using the following equation, Y=log(Y), to convert to a guassian distribution for statistical analysis. A repeated measures two-way analysis of variance, comparing each differentiation stage and each tissue source, was performed (Table 2). This analysis confirmed that the differentiation stage had an extremely significant effect on c-mpl-APC RMFI when considering all tissue sources studied (P < 0.0001). For each tissue source, a post-test for a linear trend was significant for a decreasing trend from differentiation stage I through stages II, III and IV. A Bonferroni Multiple Comparison post-test was calculated for each tissue source to individually compare each stage with every other stage. The difference in c-mpl RMFI between stage III and stage IV was not significant for any tissue source. The difference in RMFI between stage I and stage II was significant for the mPBSC tissue source only (p < 0.01). All other comparisons between the stages were significantly different in all four tissue sources studied [I vs. III, I vs IV, and II vs IV: all p < 0.001; II vs III: p < 0.001 (ABM&mPBSC), p < 0.01 UCB, p < 0.05 FL]. Similar to the % c-mpl^+ ^results, the largest contiguous decrease in the mean RMFI (transformed data) for each tissue source occurred in the transition from stage II to stage III. Thus, the greatest log decrease in c-mpl receptor density is correlated with strong expression of the CD38 surface antigen. However, the interaction between the tissue source and all values of the differentiation stage was not significant (P = 0.0779). Furthermore, the overall effect of the tissue source on c-mpl-APC RMFI was not significant (P = 0.2102) [Figure 2D].

**Table 3:** Relative MFI of c-mpl expression in human HSC/PC differentiation stages

			**Relative Median Fluorescence Intensity**			
			
**Tissue Source**	**#**		**Stage I**	**Stage II**	**Stage III**	**Stage IV**
**FL**	**5**	**Mean**	**7.13**	**3.68**	**1.39**	**1.00**
		*S.D.*	*2.06*	*1.15*	*0.40*	*0.56*
**UCB**	**5**	**Mean**	**13.14**	**8.38**	**2.71**	**1.92**
		*S.D.*	*7.77*	*6.04*	*2.03*	*1.16*
**ABM**	**5**	**Mean**	**22.25**	**9.04**	**1.34**	**0.97**
		*S.D.*	*18.32*	*6.12*	*0.38*	*0.24*
**mPBSC**	**4**	**Mean**	**26.55**	**5.72**	**0.94**	**0.47**
		*S.D.*	*36.08*	*5.01*	*0.29*	*0.14*

ABM, adult bone marrow; FL, fetal liver; mPBSC, cytokine-mobilized peripheral blood stem cells; SD, standard deviation; MFI, median fluorescence intensity; HSC/PC, hematopoietic stem cells/progenitor cells; UCB, umbilical cord blood

**Figure 3:**
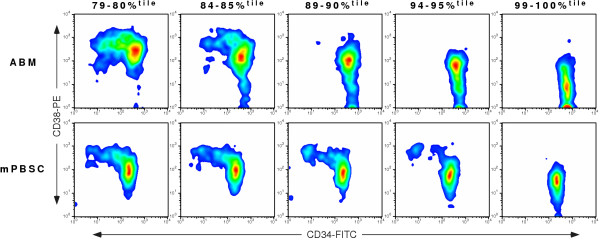
**The intensity of c-mpl expression can define the most primitive HSC**. CD34-FITC vs. CD38-PE smoothed pseudocolor dot plots derived from the same representative human CD34^+ ^ABM and mPBSC specimens as depicted in Fig. 1A. From each specimen, all cell events were initially categorized by their percentile level of fluorescence on the c-mpl-APC parameter on a log histogram plot. Five gates were drawn on the histogram plot to define a progression of five discrete levels of High intensity c-mpl expression: 79–80, 84–85, 89–90, 94–95, and 99–100 percentiles. Viable cell events falling within each percentile range are depicted on the corresponding smoothed dot plot.

We also sought to determine whether those individual cells expressing the very highest levels of c-mpl receptor, as measured by fluorescence intensity of the log APC parameter, were clustered together within a discrete group, when considering the parameters CD34-FITC and CD38-PE. Furthermore, we sought to determine whether individual cells expressing the lowest levels of c-mpl receptor could similarly be characterized in terms of CD34-FITC and CD38-PE. This would allow us to determine, in a qualitative sense, whether the density of expression of the c-mpl receptor on an individual cell could define that cell in terms of CD34 and CD38 antigen expression. In every sample studied, virtually all of the cells falling within the 98–100%^tile ^c-mpl-APC fluorescence intensity were clustered among those cells expressing the highest levels of CD34 and the lowest levels of CD38 (Figure 1C&D). This was true regardless of the tissue source of the HSC/PCs. Not surprisingly, these high intensity c-mpl expressing cells had a narrowly defined low FSC and low SSC, with minimal spread (data not shown). The cells falling within the 18–20%^tile ^of c-mpl-APC fluorescence intensity were more loosely clustered among those cells expressing a wide range of levels of CD34 surface antigen, from low to moderate to high, as well as a range of CD38 surface antigen, from moderate to very high, reflecting the inherent heterogenity of this population. The vast majority of these low intensity c-mpl-expressing cells expressed high to very high levels of CD38 surface antigen. Interestingly, few of these low intensity c-mpl-expressing cells expressed very low levels of CD38 surface antigen. Among the cells that expressed high CD38 antigen density, c-mpl^Bright ^cells were very rare. In other words, there was very little overlap between the high intensity and low intensity c-mpl stained cells on a CD34-FITC vs. CD38-PE contour plot, and virtually no overlap between the two populations at both the high and low extremes of CD38 surface antigen expression. This was true regardless of the tissue source of the HSC/PCs studied. As expected, the FSC and SSC profile of these low intensity c-mpl stained cells exhibited a wider and higher range of scatter than the high intensity c-mpl stained cells (data not shown). Not surprisingly, those cells expressing an intermediate level of c-mpl surface receptor expressed a broad range of CD34 and CD38 levels that fell within the extremes displayed by the high and low intensity c-mpl stained cells. Importantly, these intermediate level c-mpl receptor-expressing cells did not overlap with the high level c-mpl expressors at the lowest range of CD38 antigen expression. In fact, the analysis of a select number of specimens for a more comprehensive series of High intensity c-mpl expressors (79–80%^tile^, 84–85%^tile^, 89–90%^tile^, 94–95%^tile^, and 99–100%^tile^) indicated that the CD34^+ ^cells that express the very lowest levels of CD38 antigen were nearly exclusive to the cells with the very highest intensity c-mpl-expression (Figure 3).

### The CD34^+^CD38^--/dim^c-mpl^+ ^subset is capable of thymic reconstitution

**Figure 4:**
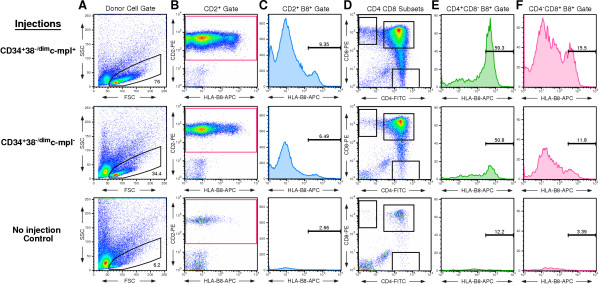
**Human c-mpl^+ ^HSC/PC have increased T-lineage repopulation capability compared with the corresponding c-mpl^-- ^HSC/PC**. Small 1 mm^3 ^fragments of human HLA-B8^-- ^fetal thymus and fetal liver were co-implanted, adjacent to one another, under the renal capsule of *scid/scid *(SCID) mice, generating one SCID-hu Thy/Liv graft per mouse. Viable, robust 8-week old Thy/Liv grafts (n = 21) were exposed to sublethal irradiation (400 Rads) in vivo, then injected with either 60,000 or 30,000 sorted human HLA-B8^+ ^CD34^+^CD38^--/dim^c-mpl^+ ^or CD34^+^CD38^--/dim^c-mpl^-- ^ABM cells and allowed to engraft for 8 weeks. Three grafts were reserved as non-injected controls. After 8 weeks, the Thy/Liv grafts were harvested, and single cell suspensions prepared from these grafts were stained with PI and a panel of fluorescent-conjugated antibodies to T-lineage markers and HLA-B8, with all appropriate isotype controls. 100,000 events from each graft were acquired on a FACSCalibur™ flow cytometer. Representative examples of CD34^+^CD38^--/dim^c-mpl^+^-injected grafts (n = 4), CD34^+^CD38^--/dim^c-mpl^--^-injected grafts (n = 7), and non-injected grafts (n = 3) are shown. **A**. Using FlowJo^® ^software, a pseudocolor dot plot of Forward Scatter vs. Side Scatter was generated and a Human Lymphoid Cell Gate was applied to isolate engrafted human mononuclear cell (MNC) events. **B**. Viable (PI^--^) human MNC events were then plotted on CD2-PE vs. HLA-B8-APC dot plot, and a CD2-PE^+ ^gate was applied to isolate all viable CD2-PE^+ ^MNC events. **C**. The total CD2-PE^+ ^MNC events were next plotted on an HLA-B8-APC histogram plot to apply HLA-B8^-- ^and HLA-B8^+ ^gates. The HLA-B8^-- ^and HLA-B8^+ ^gates were generated from the three non-injected Thy/Liv grafts combined. The HLA-B8^-- ^gate was defined to include 99.0% of the left-most viable MNC events of the non-injected grafts on an HLA-B8-APC log histogram plot. The HLA-B8^+ ^gate was drawn from the end of the HLA-B8^-- ^gate to the far right of the histogram plot. The percentage of viable (PI^--^) CD2-PE^+ ^HLA-B8^+ ^events, out of the total viable CD2-PE^+ ^MNC population, is shown for the representative examples of the three treatment arms. **D**. CD4-FITC vs. CD8-PE pseudocolor dot plot of viable (PI^--^) Human Lymphoid Cell gated events. Gates were applied to depict the thymocyte subsets (CD4^+^CD8^+^, CD4^+^CD8^--^, CD4^--^CD8^+^). **E**. Total viable (PI^--^) CD4^+^CD8^-- ^MNC events were plotted on an HLA-B8-APC log histogram plot and the HLA-B8^+ ^gate was applied. The percentage of viable donor-derived CD4^+^CD8^--^HLA-B8^+ ^events out of the total viable CD4^+^CD8^-- ^MNC events is shown for the representative examples of the three treatment arms. **F**. Total viable (PI^--^) CD4^--^CD8^+ ^MNC events were plotted on an HLA-B8-APC log histogram plot, the HLA-B8^+ ^was applied, and the percentage of viable, donor-derived CD4^--^CD8^+^HLA-B8^+ ^MNC events out of the total viable CD4^--^CD8^+ ^MNC population in the Thy/Liv graft was calculated.

**Figure 5:**
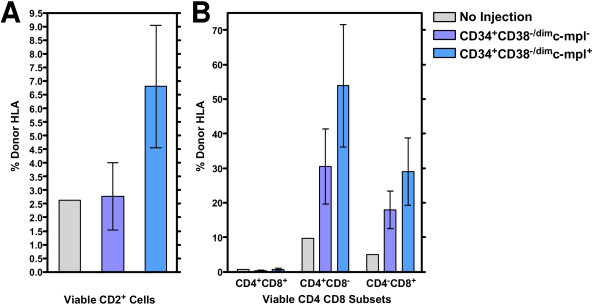
**Human c-mpl^+ ^HSC/PC show significant repopulation of mature single positive CD4^+ ^and CD8^+ ^thymocytes**. SCID-hu Thy/Liv grafts (HLA-B8^--^; n = 21) were sublethally irradiated and injected with 60,000 or 30,000 sorted HLA-disparate (HLA-B8^+^) CD34^+^CD38^--/dim^c-mpl^+ ^or CD34^+^CD38^--/dim^c-mpl^-- ^human ABM cells and allowed to repopulate for 8 weeks. Three grafts were reserved as non-injected controls. The surviving Thy/Liv grafts (c-mpl^+ ^= 4; c-mpl^-- ^= 7; Controls = 3) were harvested and single cell suspensions were stained with PI and fluorescence-conjugated antibodies against T-lineage markers, the HLA-B8 donor marker, and appropriate isotype controls. Events (100,000) were acquired and analyzed using FlowJo^® ^software to determine the percentage of viable (PI^--^) donor-derived (HLA-B8^+^) MNC expressing the different T-lineage markers from among all viable MNC expressing that particular marker. Viable (PI^--^) MNC events from the non-injected Thy/Liv grafts were analyzed to establish the HLA-B8^-- ^gate (representing 99.0% of the left-most events on an HLA-B8-APC log histogram plot) and the HLA-B8^+ ^gate (extending from the end of the HLA-B8^-- ^gate to the far right of the log histogram). These gates were then applied without alteration to the HLA-B8-APC log histogram plots of thymocyte subsets from the c-mpl^+ ^and c-mpl^--^-injected Thy/Liv grafts. **A**. The mean percentage of viable (PI^--^) CD2-PE^+ ^HLA-B8^+ ^events, out of the total viable CD2-PE^+ ^MNC population is shown for the non-injected grafts (n = 3), the CD34^+^CD38^--^c-mpl^--^-injected grafts (n = 7), and the CD34^+^CD38^--^c-mpl^+^-injected grafts (n = 4). Error bars = ± SEM. **B**. The mean percentage of viable (PI^--^) HLA-B8^+ ^donor-derived CD4/CD8 subset thymocytes [CD4^+^CD8^--^, CD4^--^CD8^+^, CD4^+^CD8^+^, CD4^--^CD8^--^], out of the respective total thymocyte subset population, is shown for the three treatment arms. Error bars = ± SEM.

One limitation of the SCID-*hu *bone model of human hematopoiesis [20] is that it lacks the thymic microenvironment necessary for the development and maturation of the T-lymphoid compartment. In order to compare the T-lymphoid reconstitution potential of human CD34^+^CD38^--/dim^c-mpl^+ ^cells versus that of CD34^+^CD38^--/dim^c-mpl^-- ^cells, we used the SCID-*hu *Thy/Liv model of human hematopoiesis [25-28]. Thy/Liv grafts were directly injected with 60,000 or 30,000 HLA-disparate human ABM cells of the CD34^+^CD38^--/dim^c-mpl^+ ^or the CD34^+^CD38^--/dim^c-mpl^-- ^phenotype. Three Thy/Liv grafts were set aside as non-injected controls. The injected cells were allowed to repopulate the Thy/Liv grafts for 8 weeks. After 8 weeks, the Thy/Liv grafts were harvested and single cell suspensions prepared. Donor repopulation (HLA-B8) of the thymic compartment was assessed by flow cytometry with the following antibody panels: (1) CD34-FITC, CD2-PE, and Donor HLA-B8-APC; and (2) CD4-FITC, CD8-PE, and Donor HLA-B8-APC (Figure 4A–F). Overall, the CD34^+^CD38^--/dim^c-mpl^+ ^injected cells showed donor-derived T-lymphoid repopulation in 75% (3 out of 4 grafts) of the mice, whereas the CD34^+^CD38^--/dim^c-mpl^--^-injected cells, demonstrated T-lymphoid repopulation in 57% (4 out of 7 grafts) of the mice. The mean percentage contribution of donor-derived cells to the stages of human T-cell development were also compared for grafts injected with CD34^+^CD38^--/dim^c-mpl^+ ^or CD34^+^CD38^--/dim^c-mpl^-- ^versus the non-injected control grafts (Table 4). For the early stage thymocytes (double positive: CD4^+^CD8^+^), both injected populations showed very low HLA-B8 expression levels that were not significantly different from the non-injected controls. Presumably, low HLA expression in these immature thymocytes limited our ability to discern the identity of donor cells in this population. For the total (CD2^+^) and the mature (single positive: CD4^+^CD8^--^, CD4^--^CD8^+^) T-cell development stages, the mean percentage contribution of HLA-B8^+ ^donor repopulation was higher in the CD34^+^CD38^--/dim^c-mpl^+ ^injected grafts than in the grafts that received CD34^+^CD38^--/dim^c-mpl^-- ^cells (Figure 5). A one-way analysis of variance was performed for each T-cell subset comparing the CD34^+^CD38^--/dim^c-mpl^+ ^and CD34^+^CD38^--/dim^c-mpl^--^-injected grafts with the non-injected grafts (Table 5). This analysis found the means of the three treatment arms to be significantly different in the CD2^+^, CD4^+^CD8^--^, and CD4^--^CD8^+ ^subsets (P < 0.05). A Dunnett's Multiple Comparison post-test was then performed for the subsets showing significant differences, comparing separately the c-mpl^+ ^and c-mpl^--^-injected grafts with the non-injected control grafts. For these subsets, the difference in means between the c-mpl^+^-injected grafts and the control grafts was significant (P < 0.05), whereas the difference in means between the c-mpl^--^-injected grafts and the control grafts was not significant (P > 0.05). Furthermore, a post-test for a linear trend from non-injection control to c-mpl^-- ^injections to c-mpl^+ ^injections was significant (P < 0.01). Thus, the increase in the mean percentage of donor-derived cells noted for each subset when moving in the order of control grafts to c-mpl^--^-injected grafts to c-mpl^+^-injected grafts appears meaningful. This analysis found no significant difference in the degree of donor repopulation between injections of 60,000 or 30,000 cells.

**Table 4:** Thymocyte Repopulation of SCID-hu Thy/Liv Grafts

**HLA^+ ^Donor Cell Transplant**	**Non-injected Control**	**CD34^+^CD38^--/dim^c-mpl^--^**	**CD34^+^CD38^--/dim^c-mpl^+^**
**No. Mice Engrafted**	**NA**	**4/7**	**3/4**

**% Donor CD2+**	**2.66**	**2.80 **(*3.27*)	**6.83 **(*4.48*)
**% Donor CD4+8+**	**0.82**	**0.48 **(*0.47*)	**0.92 **(*0.64*)
**% Donor CD4+8-**	**9.84**	**30.7 **(*28.9*)	**54.1 **(*35.4*)
**% Donor CD4-8+**	**5.17**	**18.1 **(*14.3*)	**29.2 **(*19.4*)

HLA, human leukocyte antigen; SCID-hu Thy/Liv, *scid/scid *mouse with human thymus/liver implants

**Table 5:** One Way ANOVA of SCID-hu Thy/Liv Thymocyte Repopulation

***Thymocyte Subset***	**One way ANOVA**	**Dunnett's Multiple Comparison Post-Test**		**Linear Trend Post-Test**
			
		**c-mpl^+ ^vs. control**	**c-mpl^-- ^vs. control**	
**CD2+**	P < 0.05	P < 0.05	NS	P < 0.05
**CD4+8+**	NS	NS	NS	NS
**CD4+8-**	P < 0.05	P < 0.05	NS	P < 0.001
**CD4-8+**	P < 0.05	P < 0.01	NS	P < 0.001

Values represent calculated p values for the indicated analyses. ANOVA, analysis of variance; NS, not significant (p > 0.05); SCID-hu Thy/Liv, *scid/scid *mouse with human thymus/liver implants

## Discussion

The marker of choice in human clinical HSC/PC selection strategies has generally been CD34 [3,29-32]. However, clinical selection strategies utilizing other potentially more selective HSC markers, such as CD133 [1] or CDCP1 [7], have been proposed. While the consensus phenotype of primitive human HSC has been CD34^Bright ^CD38^--/dim ^HLA-DR^--/dim ^CD90^+ ^CD117^+ ^Lineage^-- ^and rhodamine123^lo^, this report indicates that c-mpl^Hi/Bright ^may reasonably be included as a defining characteristic of primitive human CD34^+ ^HSC. We found for all tissue sources that c-mpl expression is highest in the most primitive category of CD34^+ ^HSC/PC defined by Terstappen et al. [6], then decreases significantly as the HSC/PC differentiation stage matures, manifested by increasing levels of the CD38 differentiation antigen. The greatest and most significant decrease in % c-mpl^+ ^cells occurs from stage II to stage III for all tissue sources. Similarly, the greatest decrease in c-mpl antigen levels, as measured by Relative MFI, occurs from stage II to stage III, indicating a close inverse relationship between c-mpl and CD38 antigen expression.

One potential advantage of using c-mpl as an identifier of human HSC is the known biologic role of its ligand partner, TPO. Borge et al. [33] observed that TPO promoted the viability of 51% of multifactor-responsive human CD34^+^CD38^-- ^ABM HSC/PC, 75% of which remained viable in the absence of detectable cell proliferation. This was significantly higher than that of IL-3 (25%), c-kit ligand (14%), or Flt-3L (14%). In contrast to the potent ability of TPO to promote viability of CD34^+^CD38^-- ^HSC/PC, TPO did not promote the viability of CD34^+^CD38^+ ^HSC/PC. Up to 38% of the individual CD34^+^CD38^-- ^HSC/PC surviving in 116 hours of serum-free preincubation medium containing a single cytokine, TPO, were multipotent, generating myeloid, erythroid, and mixed myeloid/erythroid colonies in methylcellulose containing a 10-cytokine cocktail. Although the studies did not assay for the lymphoid compartment, our results suggest that these TPO-responsive CD34^+^CD38^-- ^single cells possess both B-lymphoid [20] and T-lymphoid potential as well.

Previously, using SCID-hu Bone repopulation assays, we showed that the capacity for CD34^+ ^HSC/PC repopulation correlates with the c-mpl^+ ^fraction of CD34^+^CD38^-- ^human ABM HSC [20]. These results are consistent with the work of Petzer et al. [12] who showed that TPO could expand LTC-IC using an *in vitro *assay to detect very primitive HSC/PC. Borge et al. [33] subsequently demonstrated that single CD34^+^CD38^-- ^cells pre-incubated in serum-free medium containing TPO alone for 116 hours retained nearly all [96%] of the LTC-IC capacity present in single CD34^+^CD38^--^cells freshly placed in the human BM stroma LTC-IC culture assay. Interestingly, while primitive human HSC/PC have been posited to express low levels of c-kit, significantly more LTC-IC survived in the presence of TPO alone than in the presence of c-kit ligand alone. The authors also observed that, in contrast to TPO, most of the viability-promoting effect of the IL-3, c-kit ligand, or Flt-3L cytokines individually on CD34^+^CD38^-- ^cells was associated with cell proliferation. They theorize that the CD34^+^CD38^-- ^cells that proliferate in response to individual cytokines possibly represent a more mature progenitor subpopulation than those that survive without undergoing cell division. It is possible that this more mature subpopulation that proliferates in response to cytokines could represent the CD34^+^CD38^--^c-mpl^-- ^HSC/PC fraction. Because the CD34^+^CD38^-- ^subset represents a heterogeneous population, assaying for c-mpl antigen density using selective monoclonal antibodies such as CD110 (clone 3G4) [21,22] may offer a practical method to select a more primitive HSC/PC within the subset from any tissue source, potentially representing a high percentage of the long-term multilineage repopulating capacity of CD34^+^CD38^-- ^HSC/PC [33]. Importantly, we found for all tissues, that the intensity of c-mpl expression can define a relatively homogeneous population of CD34^+ ^MNC with high CD34 and very low CD38 expression.

Studies of patients with congenital amegakaryocytic thrombocytopenia (CAMT) point to the essential role of c-mpl^+ ^HSC subsets in maintaining human long-term hematopoiesis *in vivo*. The pathophysiology of CAMT was recently determined to be the result of mutations in the c-*mpl *gene, leading to defective responsiveness to TPO [34]. Ballmaier et al. found that the percentage of CD34^+ ^BM-MNC in younger CAMT patients near their time of diagnosis was within the range of normal age-matched controls, but progressively and consistently declined as patient age increased [34,35]. Furthermore, the CD34^+ ^BM-MNC from CAMT patients showed a diminished potential to form myeloid, erythroid and megakaryocytoid CFU *in vitro *compared to normal controls, and this reduced potential was accentuated with increasing age. Recently, Koka et al. [36] reinforced our previous *in vivo *SCID-hu Bone repopulation study [20] by finding that erythroid, myeloid and megakaryocytoid *in vitro *colony-forming activity (CFA) of secondarily engrafted human CD34^+ ^HSC/PC exposed to human immunodeficiency virus type 1 (HIV-1) infection in primary SCID-hu Thy/Liv grafts correlated with c-mpl expression. They also found that secondary erythroid, myeloid and megakaryocytoid CFA *in vitro *was inhibited by blocking antibodies to c-mpl administered *in vivo *to human HSC/PC residing within primary SCID-hu Thy/Liv grafts. Furthermore, a recent report documents a patient with Systemic Sclerosis and an autoantibody to c-mpl who developed pancytopenia, providing a naturally occurring example of a blocking antibody to c-mpl and its resultant inhibitory impact on human multilineage hematopoiesis *in vivo *[37].

Studies have suggested the existence of HSC from select tissue sources in early fetal life that are intrinsically different [38-40] and more primitive than HSC isolated from various tissue sources in later development and adult life [41]. We found no significant differences in c-mpl expression between the different tissue sources of HSC. Several authors have found a greater proportion of immature (CD38^-- ^and/or HLA-DR^--^) CD34^+ ^cells in UCB compared to the other tissue sources, in which no differences were noted [42-51]. Sovalat et al. [42] found that UCB CD34^+ ^cells had a significantly higher percentage of the CD34^+^CD38^-- ^subset than did CD34^+ ^cells from normal ABM, mobilized ABM, or mPBSC. The percentage of CD34^+ ^HSC/PC from normal PB that were CD38^-- ^was significantly lower than in CD34^+ ^cells from all other tissue sources. While we did not address this question directly, our study examining differentiation stage subsets raises the possibility that differences in stem cell capacity between tissue sources may be explained by differences in the quantity of c-mpl^Bright/+ ^HSC residing within the tissue, rather than by qualitative differences in c-mpl antigen expression of HSC.

Henon et al. [52,53] found that the CD34^+^CD38^-- ^HSC/PC subset was fundamentally important for early and late multilineage engraftment following autologous mPBSC transplantation. They found no correlation with infused numbers of MNC, CD34^+^CD33^-- ^cells or CD34^+^CD33^+ ^cells, and weak, inconsistent correlation of CFU-GM, CD34^+ ^cells or CD34^+^CD38^+ ^cells, with the number of days to reach predetermined recovery parameters of absolute neutrophil count, platelet count and reticulocyte count. Although previous studies have defined a CD34^+ ^cell threshold value – above which reliable and rapid engraftment was stated to occur – actual demonstration of a linear correlation between infused CD34^+ ^cells and neutrophil engraftment was rarely reported [52]. Indeed, in their study, the use of a CD34^+ ^threshold value as a predictive factor for engraftment was limited: in 24% (11/45) of their patients, the CD34^+ ^content was not predictive for rapid hematopoietic recovery. The authors discovered, rather, that the content of CD34^+^CD38^-- ^cells in the graft products was the strongest and most reliable predictive factor for both short-term and long-term tri-lineage hematopoietic engraftment. Our study provides evidence for the theory that this is explained mechanistically by the high level of c-mpl^+ ^expression within this stage I mPBSC subset.

Henon and colleagues found that a threshold value of 5 × 10^4 ^CD34^+^CD38^-- ^cells/kg BW is a reliable tool to predict engraftment [52]. Patients that received >5 × 10^4 ^CD34^+^CD38^-- ^cells/kg BW experienced a significantly faster engraftment of neutrophils, platelets, and reticulocytes than those that received less. Moreover, pre-transplant total body irradiation conditioning regimens significantly prolonged platelet and reticulocyte recovery kinetics in only those patients whose CD34^+^CD38^-- ^cell dose was <5 × 10^4 ^CD34^+^CD38^-- ^cells/kg BW. The authors also found that post-transplant administration of hematopoietic growth factors was beneficial for accelerating tri-lineage engraftment in only those patients who received a CD34^+^CD38^-- ^cell dose <5 × 10^4 ^cells/kg BW. Similarly, post-transplant rh-G-CSF administration reduced the length of hospitalization and post-transplant costs in only those patients whose mPBSC infusion contained <5 × 10^4 ^CD34^+^CD38^-- ^cells/kg BW [54]. In the setting of allogeneic BMT for the treatment of leukemia or lymphoma, Waller et al. [55] found that the CD34^+^CD38^-- ^cell content of the graft product was the best predictor for time to reach the recovery parameters of absolute neutrophil count ≥1 × 10^9^/L and platelet count ≥20 × 10^9^/L. Based on our current report and previous work [20], further studies are warranted to determine whether the relevant cell type within the CD34^+^CD38^-- ^subset identified by Henon, Waller and colleagues as capable of rapid, sustained, tri-lineage engraftment of both autologous mPBSC and allogeneic ABM transplants is the c-mpl^Bright/+ ^cell.

Fox et al. [56-58] elucidated a critical role for TPO in murine HSC engraftment following BMT. Five-fold more murine ABM cells were needed to re-establish hematopoiesis following lethal irradiation in *tpo*^-/- ^mice versus *tpo*^+/+ ^mice. Thus, TPO appears to exert a short-term radioprotective effect, presumably by enhancing the survival of the c-mpl^+ ^(and based on our study, predominantly CD34^+^CD38^--^) HSC/PC residing in the tissue. This short-term radioprotective effect of TPO on its ligand-expressing HSC/PC may explain the negative impact of total body irradiation on patients who received very low doses of CD34^+^CD38^-- ^cells in their mPBSC transplants, as observed by Henon and colleagues [52]. Furthermore, there was an *in vivo *expansion of 10–20-fold more long-term repopulating HSC in the primary *tpo*^+/+ ^recipients versus the primary *tpo*^--/-- ^recipients. These results suggest that the murine ABM c-mpl^+ ^HSC subset contains HSC that provide both short-term radioprotection and long-term repopulation following BMT. Studies in mice have also shown that *in vivo *[59] or *ex vivo *[60] priming of donor ABM cells with TPO can accelerate the reconstitution of platelets and red cells and ameliorate post-transplant thrombocytopenia.

Murine TPO mRNA is expressed by RT-PCR in embryonic stem cells, whereas murine c-mpl mRNA is expressed in the embryonic yolk sac, at day 3 of embryoid body *in vitro *differentiation, and in the blast-colony forming cell, an *in vitro *surrogate for the hemangioblast common precursor to the endothelial and hematopoietic lineages [61]. These findings indicate a potential role for the TPO/c-mpl pair in maintaining the self-renewal potential of the hemangioblast. It will be important to determine whether c-mpl is expressed in the recently identified murine embryo primitive streak-derived *brachyury*^+ ^*Flk-1*^+ ^cell population, within which the hemangioblast resides [62].

## Conclusion

This report demonstrates that human c-mpl surface receptor is expressed at the highest density on the most primitive subset within the human CD34^+ ^HSC/PC population, regardless of the tissue source or stage in ontogeny. In addition, these c-mpl^+ ^HSC/PC can also provide for thymic reconstitution and T-cell lineage repopulation in a manner that is at least as robust as, if not more robust than, that of the c-mpl^-- ^progenitor subset. Our results reported here, combined with our previous studies of c-mpl^+ ^versus c-mpl^-- ^competitive HSC repopulation in murine and human model systems [20], building on the investigations of TPO by others [11,12,33,63], suggest that c-mpl is a selective, practical and physiologically relevant marker of human long-term multilineage repopulating HSC. It has advantages in being more selective than CD34, its function relevant to HSC biology is more fully characterized than candidate markers such as CD133 [64-67] or CDCP1 [7,8], and isolation strategies may be less toxic and technologically simpler than DNA binding dyes to identify side population cells [68], or fluorescent substrates to identify aldehyde dehydrogenase^Hi ^cells [69,70]. Studies are indicated to evaluate whether c-mpl^Bright/+ ^cell number may be a superior measure for monitoring mobilization levels of peripheral blood for the timing and optimization of apheresis collections, and a more reliable and predictive gauge of stem cell content prior to transplant or following expansion. Positive selection protocols using monoclonal antibodies to c-mpl could also prove to be useful and efficient purification methodologies for stem cell banking and solid tumor purging or T-cell depletion of stem cell products.

## List of abbreviations

7-AAD, 7-amino-actinomycin D; ABM, adult bone marrow; AgD, antigen density; ANOVA, analysis of variance; APC, allophycocyanin; BM, bone marrow; BMT, bone marrow transplantation; BW, body weight; CAMT, congenital amegakaryocytic thrombocytopenia; CD, cluster of differentiation; CFA, colony-forming activity; CFC, colony-forming cells; CFU, colony-forming units; FITC, fluorescein isothyocyanate; FL, fetal liver; FSC, forward scatter; G-CSF, granulocyte-colony stimulating factor; HIV-1, human immunodeficiency virus type 1; HLA, human leukocyte antigen; HSC, hematopoietic stem cell(s); HSC/PC, hematopoietic stem/progenitor cell(s); LTC-IC, long term culture-initiating cell(s); MB, MACS^® ^Buffer; MFI, median fluorescence intensity; MNC, mononuclear cells; NC, nucleated cells; NOD, non-obese diabetic; PB, peripheral blood; mPBSC, cytokine-mobilized peripheral blood stem cells; PBS, phosphate-buffered saline; PE, phycoerythrin; PI, propidium iodide; QIIF, quantitative indirect immunofluorescence; RMFI, Relative MFI; RT-PCR, reverse transcriptase-polymerase chain reaction; SCID, severe combined immunodeficiency (*scid/scid*) mouse; SCID-hu Thy/Liv, *scid/scid *mouse with human thymus/liver implants; SD, standard deviation; SED, super-enhanced Dmax subtraction; SEM, standard error of mean; SM, staining medium; SP, side population; SSC, side scatter; TPO, thrombopoietin; UCB, umbilical cord blood.

## Competing interests

WGK has previously served as a consultant to Genentech, Inc. and currently receives funding from Genentech (co-developer of TPO) for the identification of novel antibody therapeutics in hematologic malignancies.

## Authors' contributions

WGK and JMN designed the study. WGK established the SCID-hu mouse colony and provided the necessary equipment and reagents. LCJ and CRC contributed to the procurement and collection of specimens. JMN performed the experiments, acquired the data, analyzed and interpreted the results, and drafted and revised the manuscript. WGK, CRC and LCJ reviewed, critiqued and contributed to the final manuscript.
